# Polarimetric imaging microscopy for advanced inspection of vegetal tissues

**DOI:** 10.1038/s41598-021-83421-8

**Published:** 2021-02-16

**Authors:** Albert Van Eeckhout, Enrique Garcia-Caurel, Teresa Garnatje, Juan Carlos Escalera, Mercè Durfort, Josep Vidal, José J. Gil, Juan Campos, Angel Lizana

**Affiliations:** 1 Grup D’Òptica, Physics Department, Universitat Autònoma de Barcelona, 08193 Bellaterra, Spain; 2 LPICM, CNRS, Ecole Polytechnique, Institut Polytechnique de Paris, 91120 Palaiseau, France; 3 Botanical Institute of Barcelona (IBB, CSIC-ICUB), 08038 Barcelona, Spain; 4 Departament de Biologia Cellular, Fisiologia & Immunologia. Facultat de Biologia, Universitat de Barcelona, 08028 Barcelona, Spain; 5 Department of Applied Physics, University of Zaragoza, Pedro Cerbuna 12, 50009 Zaragoza, Spain

**Keywords:** Imaging and sensing, Plant physiology, Stomata, Polarization microscopy

## Abstract

Optical microscopy techniques for plant inspection benefit from the fact that at least one of the multiple properties of light (intensity, phase, wavelength, polarization) may be modified by vegetal tissues. Paradoxically, polarimetric microscopy although being a mature technique in biophotonics, is not so commonly used in botany. Importantly, only specific polarimetric observables, as birefringence or dichroism, have some presence in botany studies, and other relevant metrics, as those based on depolarization, are underused. We present a versatile method, based on a representative selection of polarimetric observables, to obtain and to analyse images of plants which bring significant information about their structure and/or the spatial organization of their constituents (cells, organelles, among other structures). We provide a thorough analysis of polarimetric microscopy images of sections of plant leaves which are compared with those obtained by other commonly used microscopy techniques in plant biology. Our results show the interest of polarimetric microscopy for plant inspection, as it is non-destructive technique, highly competitive in economical and time consumption, and providing advantages compared to standard non-polarizing techniques.

## Introduction

The inherent properties of light are a significant source of information when used to probe the properties of vegetal tissues^1–17^. In microscopy imaging, it is common to prepare the samples to be studied in very thin sections to prevent multiple scattering of light by the different tissue structures, which can degrade image contrast and spatial resolution. Thin sections of tissues are in general almost transparent and very difficult to visualize if a contrast enhancement technique is not applied. Chemical staining is a very popular approach because of the chemical specificity of dyes to targeted molecules in the tissues. The phase contrast technique^13^ is a widely spread approach, which does not require any staining, and which can increase contrast of the thin sections proportionally to the optical thickness of the vegetal structures probed.

The use of polarized light to increase contrast in images used for vegetal tissue characterization is also a well-known approach. Contrast enhancement of images of plant structures is usually obtained using polarized light, through the measure of dichroism or birefringence^18–25^. Dichroism is related to the polarization-dependent absorption of light by plant structures and it is useful to detect specific molecules as well as to visualize how they are organized in a three-dimensional framework. Dichroism is successfully used in many studies devoted to reveal the organization and concentration of chloroplasts and related organelles in plant species^19,21^. Birefringence is generated either by anisotropic molecules (in general partially crystallized macromolecules) or by non-isotropic organization of non-necessarily anisotropic macromolecules. Birefringence has been successfully used to characterize birefringent macromolecules as cellulose, involved in distinct types of cell processes, such as cell development and aging^18^, production of guard cell protoplasts^19^. Birefringence has also been used to study the structure of guard cells themselves and their related stomata^20^ or to investigate the cell wall composition in phylogenetically distant groups of plants^21^, and to study the structure of trichomes^22,23^.

Polarimetric microscopes used for plant inspection are mostly optimized to measure dichroism or birefringence, but other polarimetric features, as depolarization, are usually neglected. Depolarization arises when photons with different polarization states incoherently reach the same area of the detector. In plants, depolarization is mainly caused by light scattered by cells, organelles, extracellular structures, and other elements that may be located within the tissue. For very thin preparations, scattering is usually low and depolarization effects are usually disregarded, however, optical characterization of plants is not always performed in such conditions. To date, the most used approach to account for depolarization introduced by plants is to measure the so-called degree of polarization (DoP) of scattered light^26–29^. Since DoP depends on the intrinsic characteristics of the constituents of plants it is a pertinent and informative observable of the state of a given specimen, reason why it has been used in preceding studies. However, a more general approach, Mueller polarimetry has been scarcely used in botany^24,25,30^. The latter situation is surprising compared to the extensive use and the still growing interest of Mueller polarimetry to study either human or animal tissues^31–36^.

The goal in the present manuscript is to show that Mueller polarimetry provides both, polarization-based and depolarization-based observables and that both of them can bring interesting and independent information about the physical properties and structure of vegetal tissues. Polarization-based observables can be measured with techniques other than Mueller polarimetry, however, Mueller polarimetry has the advantage compared to other experimental approaches that it provides all the polarization-based observables and the depolarization-based ones as the result of a single measurement. The present manuscript is to be read as a general presentation of imaging Mueller polarimetry applied to plants and the evident potential benefits that botanists can obtain when implementing it in their characterization routines. This paper goes beyond the simple illustration of a case study and compares polarization microscopy-related images with microscopy images obtained with state-of-the art techniques commonly used to visualize and to characterize plants. We demonstrate how polarization microscopy is an excellent tool for characterization of vegetal tissues and plant sections. It is a perfect complement, and in some cases is advantageous, to standard microscopy methods, providing the potential to expand the field of optical instrumentation for the study of plants.

## Results

**Figure 1:**
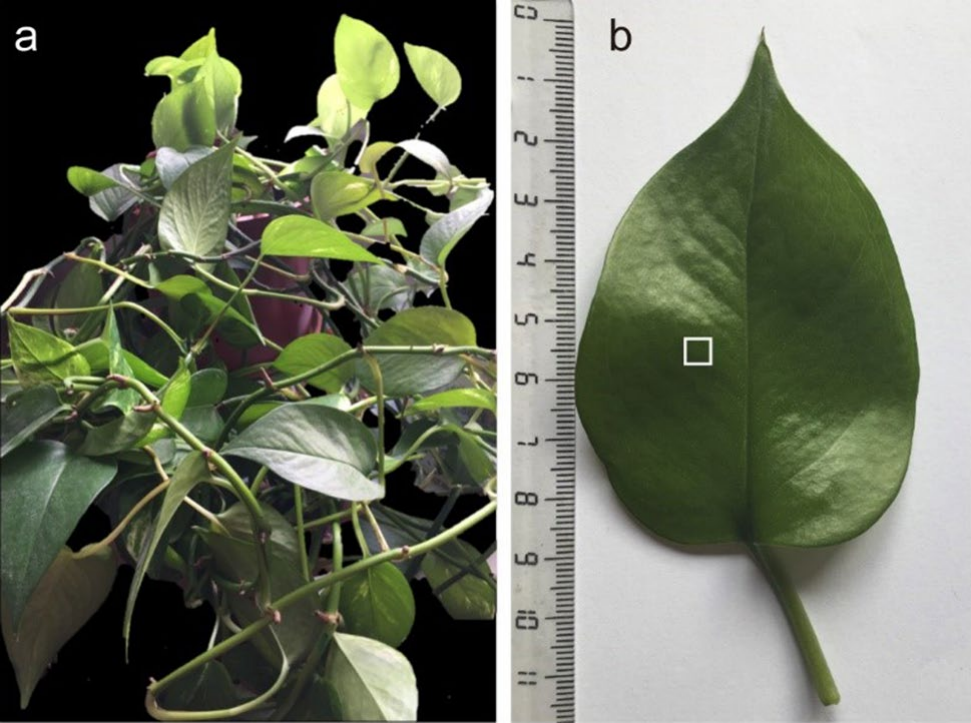
Plant specimen used for the present study: (**a**) *Epipremnum aureum* general view; (**b**) one of the measured *Epipremnum aureum* leaves.

In this work we have considered leaves from a specimen of *Epipremnum aureum* (Linden & André) G.S.Bunting belonging to the Araceae family as a case of study. A picture of said specimen is shown in Fig. 1a. Comparison of imaging polarimetry with other advanced techniques, such optical microscopy, phase contrast microscopy, fluorescent microscopy, highlights the potential of polarimetry for plant inspection. These advanced imaging methods that will be discussed in the present study are currently used in botany for plant inspection. Although the selection is not an exhaustive compilation, it is meant to be representative of the techniques used in the field and they should be interpreted here as a base of comparison to help the reader to understand the potential and the interest of Mueller polarimetry. Note that other methods could be mentioned but are not available in this study, as for instance, differential contrast microscopy (DIC) that is a relevant advanced characterization technique, and that presents images like those obtained by phase contrast microscopy but emphasizing lines and edges of the sample structures by exploiting the polarization properties of light^37^. Figure 1b shows one of the leaves used for the present study. The square inside the leave highlights the area that was imaged using the above-listed microscopic techniques. A description of the plant used for the present study is found at the Methods section.

**Figure 2:**
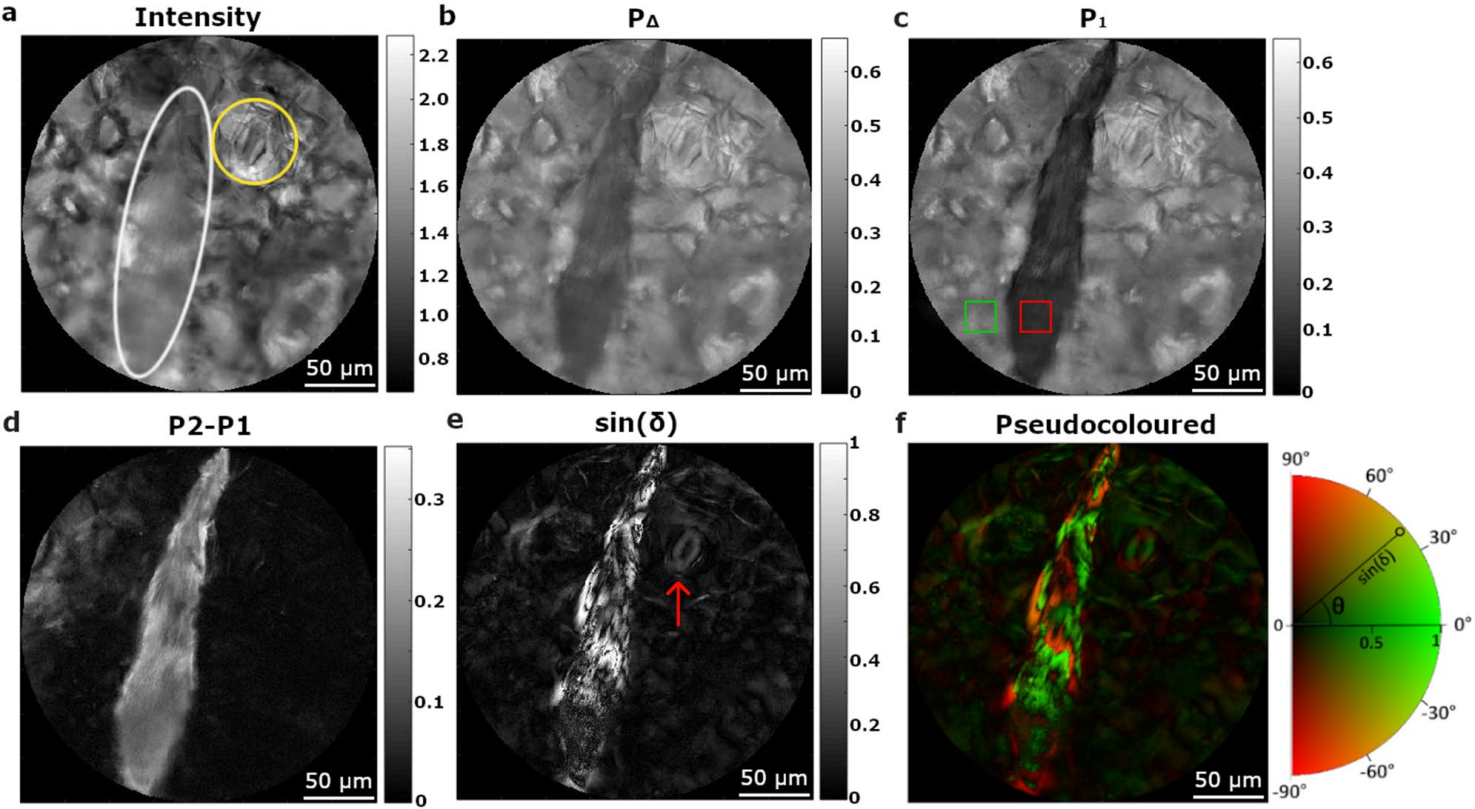
Polarimetric analysis of a small piece of the *Epipremnum aureum* leaf (marked with a white rectangle in Fig. 1b). Different images of an inulin raphide, situated close to a stoma, are provided, which were obtained by using different channels: (**a**) Intensity; (**b**) P_Δ;_ (**c**) P_1_; (**d**) P_2_–P_1_; (**e**) The sinus of linear retardance *δ* (stoma highlighted with a red arrow); and (**f**) Pseudocoloured image encoding retardance modulus and orientation. Pseudocoloured image comprises the linear retardance of the sample (shown into a white–black scale; radius of the semi-circular colour scale), and the fast axis orientation (represented with different colours; perimeter of the semi-circular colour scale).

Polarimetric microscopic images of the *Epipremnum aureum* leaves were taken with a multimodal microscope working in transmission configuration. The microscope can also be used in a way to obtain images insensitive to the polarization of light (standard optical microscope). More details of the microscope used can be found in the Methods section. An example of an image taken in non-polarized mode of the lower leaf surface of the leaf can be seen in Fig. 2a. In the latter image it is possible to distinguish the presence of a few epithelial cells, characterized by their typical polygonal shape, and a guard cell and its related stomata. These cells are located just at the lower surface of the leaf. In the image shown, there is also possible to guess the presence of an elongated structure which appears blurred because it is located inside the leaf, at a distance from the focal region longer than the depth of focus of the objective used to take the images. Because of the blurry and the lack of contrast in said image, it is not possible to perceive the details, or at least, to unambiguously identify the nature of the elongated structure. The same portion of the leaf was measured in polarimetric mode and the Mueller matrix image encoding the polarimetric response of the sample was obtained. To get further physical information from the measured Mueller matrix image, the latter was decomposed to obtain a set of subsequent images of polarization and depolarization metrics. Concerning depolarization, in this work it is used the depolarization index P_Δ_ and a set of observables (P_1_, P_2_ and P_3_) called indices of polarimetric purity (IPPs) that give indications about the way that a medium depolarizes light. Accordingly, P_Δ_ and IPPs are sensitive to classify different microscopic elements according to their ability to depolarize the illuminating light. It is worth to note that P_Δ_ is a global depolarization measure while IPP can distinguish different depolarization anisotropies that results into the same P_Δ_ value. Interested readers can found, in the Methods section and in the Supplementary information, a detailed description of the polarization-depolarization observables and an algorithm to deduce them from Mueller matrices.

Figure 2b–d show the images corresponding to P_Δ_, the first IPP, P_1_, and the difference P_2_–P_1_, which provided highly contrasted images. Concerning polarization properties, Fig. 2e shows the sinus of the linear retardance, which gives information about the birefringence of structures in the imaged area of the leaf. In Fig. 2b–e the elongated shape of an inulin raphide can be also seen. Inulin is a type of polysaccharide which crystallizes in needle-shaped crystals; the crystals tend to group together to form raphides, which are found in parenchymal cells in some plant species. In such images, the edges of the raphide can be clearly distinguished, and the whole structure is highly contrasted with respect to the background. In fact, in the case of P_1_ image (Fig. 2c), the red square section of the raphide, has an average P_1_ value of 0.11 whereas the green square section, corresponding to the background, has an average P_1_ value of 0.29. A compact structure such a raphide scatters light differently that the aqueous structure of the surrounding media. The fact that the values of P_1_ are well clustered in two groups around 0.11 and 0.29 respectively, shows the ability of the observable P_1_ to distinguish among different types of matter, which would not be possible under non-polarized light images. The case of P_2_–P_1_ channel in Fig. 2d is also exemplary. According to this observable, the same section of the raphide is characterized by values around 0.13 whereas the same section of the background cells shows the value 0.02. To quantify the image contrast between two structures in the image, the so-called visibility has been used as a metric. The visibility is defined by the expression: $V = \left| {\overline{I}_{str} - \overline{I}_{back} } \right|/\overline{(I}_{str} + \overline{I}_{back} )$, with $\overline{I}_{str}$ and $\overline{I}_{back}$ being the average signal intensities of the studied biological structure (in this case the raphide) and the background cells respectively. Visibility values are between 0 (null contrast) and 1 (maximum contrast). In the analysed case, the visibility of the image between the raphide and the background is *V* = 0.03 for the intensity image (Fig. 2a), *V* = 0.24 for P_Δ_ (Fig. 2b), *V* = 0.41 for the P_1_ observable, (Fig. 2c) and a visibility of *V* = 0.72 for P_2_-P_1_ case (Fig. 2d). Visibility values of different observables are calculated over the same red and green square sections (Fig. 2c) and further contrast analysis, including the study of the standard deviation of the intensity in these regions, is provided in the Supplementary information. Note that the visibility values of depolarizing observables are significantly better than those obtained for the polarization insensitive image. Regarding the different depolarizing observables, the visibility of the raphide is higher in the case of analysing P_1_ and P_2_–P_1_ than P_Δ_ as the raphide depolarizes light in an anisotropic way_._ Therefore, raphides are easier to identify when using these IPPs observables than in the P_Δ_ case.

Raphides are not the only structures which can be highlighted thanks to polarimetric microscopy. There are for instance guard cells and their related stomata, which can also be visualized and characterized using birefringence. Birefringence in guard cells is mostly due to a preferential alignment of small cellulose microfibrils inside their walls. Young and healthy guard cells can show regular and intense birefringent pattern, on the contrary, dead or non-functional guard cells have distended walls, and show distorted or very poor birefringence patterns. The portion of the leaf shown in Fig. 2a–f contains a stoma of about ~ 50 µm stomatal length close to the raphide. Despite of being birefringent, the visibility of the stoma is lower than that of the raphide (also birefringent) and therefore somehow screened by it in the colour scale chosen to represent Fig. 2e.

While the stoma has a negligible dichroism, the raphide is characterized by 0.2 rad of linear dichroism, which indeed appeared to be oriented along the axis of said raphide. The linear dichroism in raphide may be due to the anisotropic absorption of well aligned inulin crystals which form the raphide or to the non-isotropic scattering which attenuates differently light polarized parallel or perpendicular to the major axis of the raphide. The results of a specific study to elucidate the origin of dichroism in raphides is out of the scope of the present work but will be presented elsewhere. Moreover, the non-isotropic scattering of light due to the elongated shape of the crystals may be at the origin of the non-symmetric depolarization that gives rise to the highly contrasted P_1_ channel with respect to P_Δ_.

**Figure 3:**
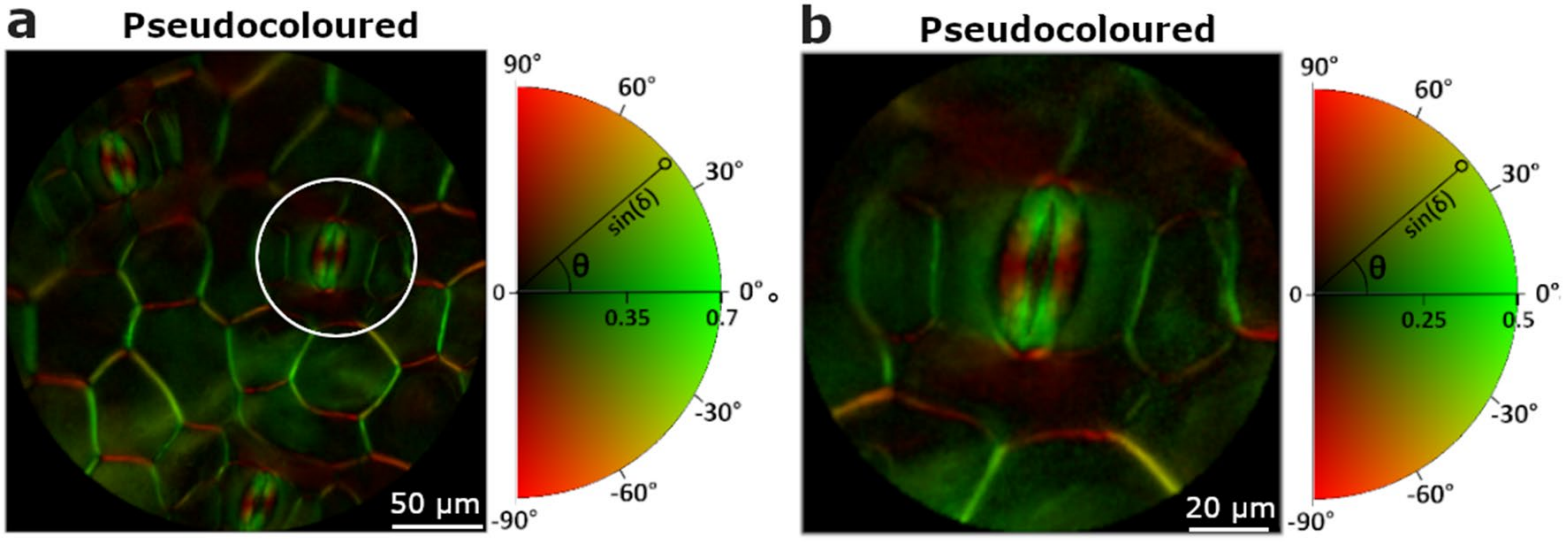
Pseudocoloured image of a collection of stomata and the zoomed image of a given stoma are shown in (**a**) and (**b**), respectively. The pseudocoloured image comprises the linear retardance information of the sample (shown into a white–black scale; radius of the semi-circular colour scale), and the fast axis orientation (represented with different colours; perimeter of the semi-circular colour scale).

A colour encoding format is an appropriate way to highlight different polarization and depolarization signatures at once in the same image^38^. For instance, Fig. 2f shows the sine of the retardance, already shown in Fig. 2e, completed with the information of the orientation of the birefringence. In a second figure, Fig. 3a, it is shown how colour encoding allows for further visualization of the stoma. The image corresponds to an area of the leaf, free of raphides, where stoma, guard cells and cell membranes are present. Colour encoding allows for a clear difference between the membranes of the guard cells and the boundaries of the stoma. The image in Fig. 3b corresponds to a zoomed view of the area encircled in Fig. 3a and shows how the structure of the stoma (the pore region) and the underlying walls of the associated guard cells can be clearly distinguished. Note that it is impossible to achieve a similar level of differentiation by using standard, non-polarimetric, visualization techniques in microscopy with unstained samples; see for instance Fig. 2a. What is more, since the colour scale in Fig. 3b is related to different orientations of the birefringence, quantitative information about strain spatial distribution can be obtained from the image^21^.

**Figure 4:**
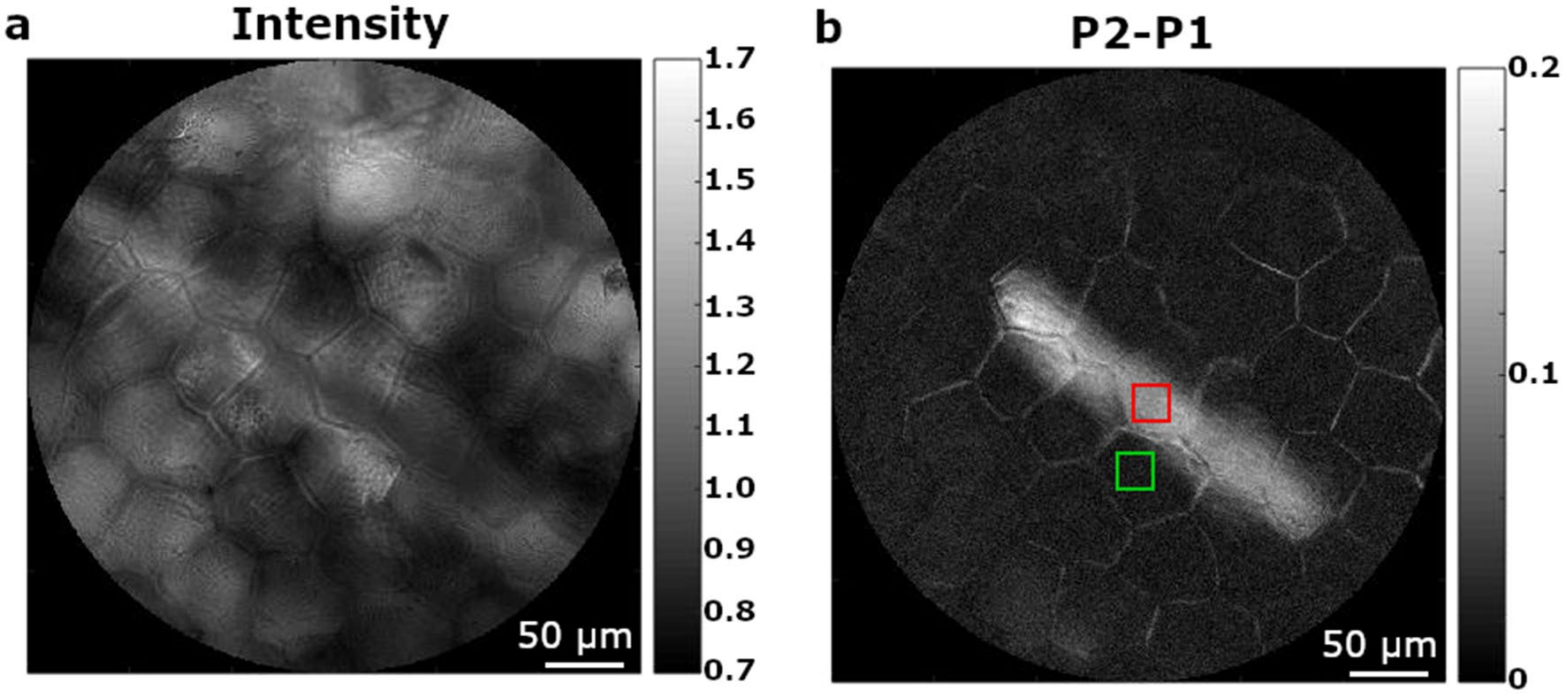
Figure (**a**) shows the intensity image of an inulin raphide out of focal plane (defocused). The corresponding image obtained by using the P_2_–P_1_ channel is provided in (**b**).

Finally, we want to highlight another relevant advantage of polarimetric imaging, the ability to highlight properties and to improve visibility of objects which in standard conditions of observations may appear blurry because they are out of focus. The latter is discussed through the following example, in which the same leaf was used, but observed with the corresponding obverse face pointing to the imaging microscope objective (the opposite face than in previously discussed examples). A region of interest was selected in which a raphide was present in the field of view, but instead of focusing on the raphide (inside the leaf) the image was focused on the surface of the leaf. Accordingly, in Fig. 4a, it is shown an image taken under unpolarized light to illustrate how the scene is viewed under standard visualization conditions. In this image, the epidermal cell walls are clearly visible and the raphide appears so out of focus and blurry that it is barely identifiable. The visibility of the raphide is *V* ~ 0 (the visibility is calculated by using the associated red and green squared regions of Fig. 4b). However, when the same region of the leaf is measured using polarimetry in identical imaging conditions, the presence of the raphide is clearly revealed in the P_2_–P_1_ channel Fig. 4b, with visibility equal to 0.67. The same image allows for the observation of the cell walls which are also contrasted respect to a black background. Cell walls and raphide are visible because both scatter light more efficiently than the bulk of the cell, and, therefore they create more light depolarization. Even though the raphide remains out of focus, it cannot be, by any means, overlooked. We think that the ability of polarimetric imaging of showing the presence of structures, even being out of focus, is a major advantage as it allows for the identification of biological structures located at different axial planes. The later permits imaging of a given region at the focal plane without loss of resolution while revealing some out of focus structures at the same time. This could be helpful to users without aprioristic information of samples, as polarimetric contrast shows to be very useful to detect relevant structures which may be out of focus.

So far, in this section we have seen the improvement associated to polarimetric imaging microscopy, with special mention to the IPPs channels, when compared with standard microscopy. However, other microscopy techniques are well-stablished in biophotonics applications. For the sake of completeness, we include here a set of images taken from the same sample specimen, the *Epipremnum aureum* leaf, obtained using two of the main optical microscopy techniques used in botanic: phase contrast microscopy and fluorescence microscopy.

**Figure 5:**
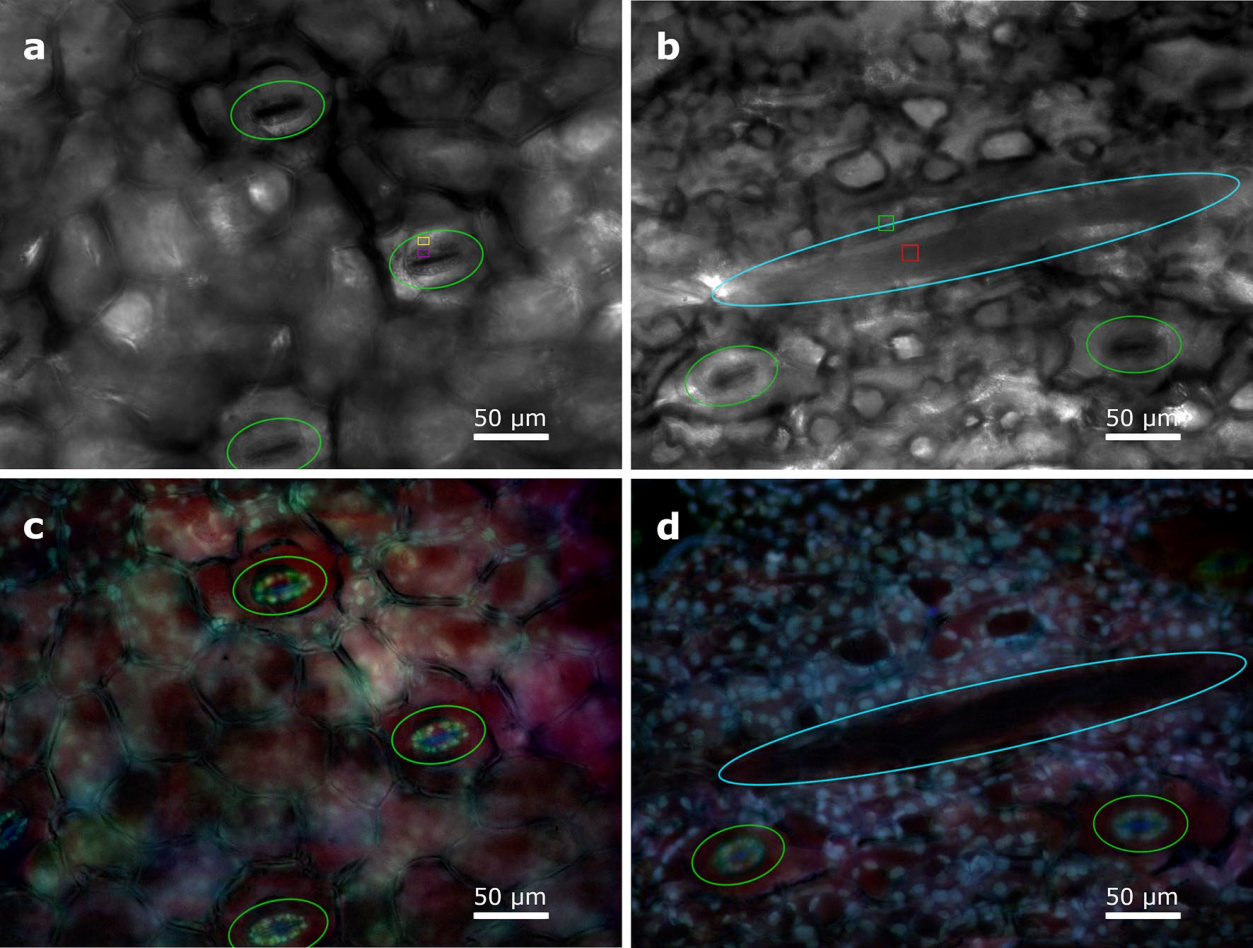
Images of *Epipremnum aureum* stomata obtained with a phase (**a**) and a fluorescence (**c**) microscope. Images of an *Epipremnum aureum* inulin raphide, obtained with the same phase and fluorescence microscopes (**b**,**d**), respectively.

Phase contrast microscopy allows for the observation of unstained cells^13^ and it is especially useful to explore living cells in real time because it does not need the evaluation of multiple images as in polarimetry. Phase contrast measures differences in the global phase of a light beam between adjacent zones in the sample under examination which are created by small variations in thickness and density (refractive index) among those zones. In phase contrast microscopy, birefringence is not needed to create a visible contrast between two areas of a sample. Phase contrast images of an almost transparent and non-contrasted object give to the human eye the impression of a shaded three-dimensional object. The latter implies a significant improvement in the perception of the sample, and it is in part what is behind the success of this technique. Representative images of stomata and raphides imaged with the phase contrast microscope are shown in Fig. 5a,b, respectively. The leaf piece was imaged using a commercial *Olympus Fluoview 1000* phase contrast microscope described in the Methods section. Whereas the stomata are clearly visible in the phase contrast microscopy image (Fig. 5a), with a visibility of V = 0.38 (V is calculated over the purple and yellow regions of Fig. 5a representing the stomata and background respectively), the raphides were no so-well contrasted, presenting a visibility reduced to V = 0.14 (V is calculated over the red and green squared regions of Fig. 5a representing the raphide and background respectively). In Fig. 5b, the location of a raphide is highlighted by a violet ellipse to help for visualization. In this example, polarimetric images produce more contrasted and more specific images than phase microscopy. Phase contrast performed less well than polarimetry, especially in the case of images of raphides, because the light scattered by raphides depends on the polarization of the incident light and phase contrast microscopy is unable to see that phenomena as it illuminates the sample with unpolarized light. Moreover, colour encoding strategies to enhance image contrast and visibility can be applied in polarimetric imaging because polarimetry consists in multiple independent channels of information whereas in phase-contrast microscopy the information is restricted to only one channel. A second reason why phase contrast performed less well than polarimetry in the example discussed here is the fact that the sample was not thin, i.e., limited to a single monolayer of cells. In these circumstances, phase shifts larger than 2π can cumulate and produce grey scales which do not linearly relate to variations in sample thickness or density, therefore degrading the performance of the technique. Phase contrast and polarimetry can be complementary because they can be used in non-stained samples. In polarimetry, some channels are specific to the manifestation of a property in the sample, such as retardation or dichroism. In this way, while phase-contrast provides an enhanced view of the tridimensional conformation of the object, polarimetric observables can highlight aspects related to certain specific properties of the sample.

Images of the same areas of the leaf explored under phase contrast imaging were taken with the same *Olympus Fluoview 1000* commercial microscope previously cited, operated in fluorescent mode. The use of specific dyes selected to link to the molecules that are of interest for the observations, makes fluorescence microscopy a highly specific and highly resolved technique. By using two different fluorescent dyes, images of well contrasted and differentiated stomata (Fig. 5c) and raphide (Fig. 5d) were obtained. Although the visualization of the raphide structure is comparable with that obtained by using polarimetric channels (Fig. 2b–d), the stoma image shows some characteristics and details which cannot be reached by polarimetric means (Fig. 5c). However, some physical information provided by polarimetric images is not present in fluorescence image, such as mechanical stress (which in turn creates birefringence) that may occur in cell walls. Polarimetric and fluorescence techniques are compatible in the sense that both can be applied to stained samples. Staining may enhance polarimetric properties, in particular dichroism, in places where the dye links to the molecules of the sample because in many cases dyes are anisotropic and dichroic. Fluorescence microscopy works well with samples prepared as very thin sections made of a monolayer of cells. For relatively thick samples, like the one used here, the light emitted by dyes can be scattered within the tissue and then to end up by degrading the spatial resolution of the images if a confocal configuration is not used. Working with thick samples is not a problem in polarimetry, provided that an adequate separation between polarization and depolarization channels can be done as shown in this work.

**Figure 6:**
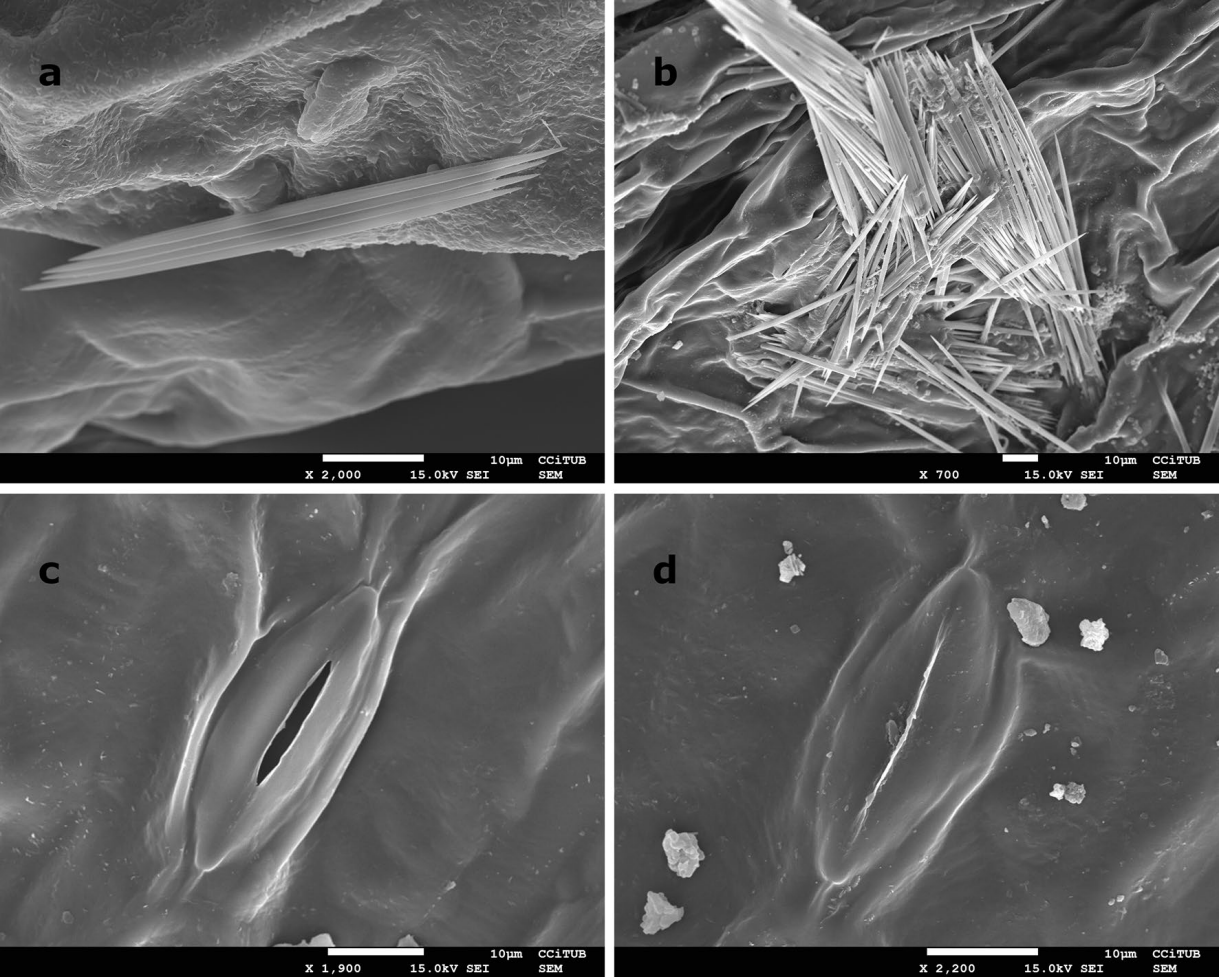
Scanning electron micrograph showing the ultrastructure of a bunch of inulin raphides (**a**) and (**b**) in the parenchyma cell of the studied *Epipremnum aureum* leaf. Stoma ultrastructure is also shown with an open (**c**) and closed (**d**) pore.

Finally, the validity of the analysis performed on the *Epipremnum aureum* leaves by polarimetric methods is confirmed by high resolution images from scanning electron microscopy (SEM) taken on the same leaves of the same plant. From SEM images, we observed a concentration of inulin raphides which are in agreement in size and shape to the structures observed in Fig. 2b–d and identified as raphides. An electron microscope image showing a representative inulin raphide in the studied *Epipremnum aureum* plant is shown in Fig. 6a. Another inulin raphide is shown in Fig. 6b, but the size of this raphide cannot be well determined because it was broken during the sample preparation. Preparing the sample without damaging the raphides is very challenging and requires a high degree of technical expertise. The images provided by SEM also confirm the presence of stomata previously shown in Figs. 2, 3 and 5. Two stomata with open and closed pores respectively are shown in Fig. 6c,d. The presence of other structures, not detected by previous methods, such as tinny salt crystals which are observed above and around the stomata pores can also be observed due to high resolution of SEM.

## Discussion

The present paper highlights the interest of polarimetric microscopy to the study of an *Epipremnum aureum* leaf, but the suitability of polarimetric methods here discussed was also observed by us in different specimens of *Hedera maroccana* McAll., *Spathiphyllum* sp*.*,* Hibiscus syriacus* L*.*,* Photinia × fraseri* Dress*, Prunus dulcis* (Mill.) D.A.Webb,* Arum italicum* Mill., *Hedera helix* L*.*, and *Vitis vinifera* L. For completeness, microscopic polarimetric images of *Heredera Helix* and *Vitis vinifera* are provided in the Supplementary information as illustrative examples. In the case of the *Epipremnum aureum*, polarimetric channels clearly show the presence of raphides and stomata in the plant. Stomata play an important role in the interaction between plants and environment^39^. These structures regulate gas exchange and water loss in plants, being both key processes in a context of increase of CO_2_ atmospheric concentration and water stress produced by extreme droughts. Despite the contrasting responses of stomata to climate change^40^, the study of these structures in living plants is especially relevant in the current scenario to determine the plant productivity by analysing its water use efficiency^41^. Measurements of stomata are typically conducted using a silicon rubber impression technique^42^ followed by a positive replica of the impression material made by using nail varnish^43^. Polarimetry is presented as a non-contact alternative technique that is faster and easier to implement. Moreover, polarimetry brings new information related with the distribution of birefringence, probably due to mechanical stain, in the stomata cell walls^21^.

Raphides are present in many plant species, their abundance and morphology (size, shape…) presence together with the crystal structure and morphology seems to be characteristic of taxonomic group of plants. These structures, which may be part of the defence mechanism of the plant due to their toxicity, are particularly frequent in the Araceae family. They are used in food and pharma industries^44,45^ and they have several medicinal applications, though they can also cause several side effects. In addition, the study of the raphides can be used in the characterization of some plant species and in their systematic classification^46^ and to inform about the toxicity of wild edible plants included in retrieval strategies^47^. Electron microscopy is routinely used to observe the crystals in detail, but the technique requires specific sample preparation. Polarimetry is presented as a non-invasive technique with an easier implementation.

The results shown in this article have been selected to illustrate the presence of different polarization and depolarization responses in plant tissues and therefore how they can be used to characterize plant sections or specific vegetal structures. Raphides are characterized by a well-defined depolarizing and dichroic response, and stomata by their retardance. These characteristic polarimetric responses allow for an easy identification of the mentioned structures (Figs. 2, 3 and 4), while being almost invisible to the most used optical instrumentation techniques. Therefore, it has been shown how polarimetric imaging provides very practical and useful tools that allow for the visualization of some plant characteristics not observed when standard non-polarized images are used. In addition, polarimetric methods can reveal some structures hidden because they are out of focus but have a distinct polarimetric response (Fig. 4). Although elaborate sample preparation, such as clearing, fixing, cutting, or mounting can of course help to improve image quality, they are not mandatory to do polarimetric imaging, which greatly simplify the sample handling and characterization procedures.

Thanks to the sensitivity of polarization to specific properties which are generally located in well-defined parts of plant, polarimetry can be complementary or even more useful than other standard characterization techniques. In fact, polarimetry can be combined with other optical techniques within the same optical instrument^35^. As an important advantage, polarimetric methods can provide unique physical information, as was the case of the non-homogeneous physical properties of the stoma revealed by polarization (Fig. 3a,b), that was hidden when using any of the other methods described in this manuscript, including electron microscopy.

The images provided by SEM (Fig. 6) confirm the results obtained by using polarimetric microscopy. Electronic images provide the best visualization of plant structures, when compared with optical techniques previously discussed. However, optical, in particular polarimetric methods are much more accessible than electronic microscope and could be used for dynamic applications (snapshot imagers). Polarimeters are based on compact (an eventually portative) optical configurations^48^, which can be used for outdoors measurements.

Summarizing, the results provided in this manuscript illustrate the potential of Mueller polarimetric microscopy for plant characterization and botanical applications, and also they illustrate the benefits of the recently devised depolarization-based observables in complement of the commonly used polarization-based ones. Mueller polarimetry provides complementary information not accessed using other optical techniques, as phase contrast or fluorescent microscopy. Electron microscopy provides images with high resolution but is less practical than polarimetry or other optical methods. Moreover, polarized light microscopy is a non-invasive technique (as it is the case of fluorescent microscopy) and can be combined with other optical techniques in the same instrument just by including very feasible setups (polarimeters) in the common path of standard optical microscope setup. In addition, some image polarimeter architectures are very compact^48^ and can be used outdoors, thus being valid to perform in situ and in vivo measurements of plants. Under this scenario, we think that Mueller polarimetry is a very interesting and promising technique to be used alone or in complement to other approaches to study plants.

## Methods

### Sample description

We measured a leaf of *Epipremnum aureum* (Linden & André) G.S.Bunting, which is a synonym of *Pothos aureus* Linden & André. This species, belonging to the Araceae family, occurs in forests from Southeast Asia to tropical Australia. The adult leaves are usually perforated and often have translucent spots along the midrib. These evergreen climbing plants are cultivated for their attractive foliage. A herbarium voucher of the studied species is deposited in the Herbarium of the Botanical Institute of Barcelona (BC843412). An image of the measured *Epipremnum aureum* is given in Fig. 1a. The leaf measured is shown in Fig. 1b.

### The Mueller matrix approach

Polarization of light is in general modified when it interacts with material media. The formalism followed in this work to describe the polarimetric modifications is the Mueller matrix approach. Within this approach, the states of polarization of light are represented by means of four real parameters, which are the components of the so-called Stokes vector. The physical meaning of the four components of the Stokes vector is related to the ellipticity, ε, and the azimuth, θ, of the polarization ellipse^49^. The polarization ellipse is the trajectory followed by the end point of the electromagnetic field when light propagates in a given media. Accordingly, the modification of the polarization state produced during light-matter interactions is described by using a 4 × 4 matrix called the Mueller matrix, in such a manner that the Stokes vector of the output light is given by the product of the Mueller matrix and the Stokes vector of the input light.

The determination of the experimental Mueller matrix requires the use of a Mueller polarimeter, which measures the polarimetric characteristics of the sample by controlling the polarization of the illumination light and analysing the state of polarization of the light eventually modified during the light-matter interaction. The determination of a Mueller matrix is obtained from a set of radiometric measurements resulting from the illumination of the sample with light prepared in different polarization states, and the subsequent analysis of the polarization of imaged (or detected) light beam. This situation is mathematically described by means of the following expression:

1 $$ {\varvec{I}} = {\varvec{S}}_{PSA} {\varvec{M}}_{Sample} {\varvec{S}}_{PSG} , $$

where ***I*** is a *n* × *n* matrix composed by the measured intensities, ***M***_*Sample*_ is the 4 × 4 Mueller matrix of the sample,* S*_*PSG*_ is a 4 × *n* matrix whose *n* columns represent the Stokes vectors of the different polarization states used to illuminate the sample, and ***S***_*PSA*_ is a *n* × 4 matrix whose rows provide the *n* different transposed Stokes vectors which represent the set of analysis polarization states over which the polarization state emerging from the sample is projected to be analyzed. The Mueller matrix can be derived from the Eq. (3) by calculating the pseudoinverse of the analyzer and the illumination matrices ($\tilde{\user2{S}}_{PSA}^{ - 1}$ and $\tilde{\user2{S}}_{PSG}^{ - 1}$) this leading to the following relation,

2 $$ {\varvec{M}}_{Sample} = \tilde{\user2{S}}_{PSA}^{ - 1} \user2{I\tilde{S}}_{PSG}^{ - 1} . $$

At least four illuminating and analyzed states are needed to measure the full Mueller matrix. Therefore, at least 16 measurements are required to fully determine ***M***_*Sample*_.

### Polarization and depolarization metrics

In the work presented here, we make use of different polarimetric metrics to analyze the optical response of vegetal samples. These metrics are calculated from the experimental Mueller matrix and they are related to the main polarimetric content of the sample, i.e., diattenuation, retardance and the degree of depolarization. Although a few metrics can be directly gathered from the Mueller matrix, such as the diattenuation *D*, other metrics can be obtained after decomposition of the Mueller matrix to a set of simpler matrices. There are different types of matrix decomposition schemes, such as product, sum and differential^50,51^ ones, each decomposition is adapted to particular and well-defined experimental conditions. Accordingly, the choice of one or another decomposition scheme must consider the experimental conditions and the sample structure. In the context of the present work a product decomposition known as Lu-Chipman decomposition^49,50^ was used to obtain the polarimetric properties from experimental data. In general, the polarimetric properties derived from different decompositions may differ to each other, the latter being due to the non-commutativity of the different algebraic operations needed to be done in order to implement the decomposition. Before doing a final choice to present the data of this work, the polarimetric data obtained with the Lu-Chipman decomposition, the symmetric decomposition and the differential decomposition were compared. For the case analysed in this article it was found that all the decompositions tested provided equivalent results. The choice of the Lu-Chipman decomposition was done because it provided slightly better results in terms of numerical noise compared to the symmetric decomposition, and because it can be applied for measurements in transmission and reflection configurations, contrarily to the differential decomposition, only valid for measurements in transmission.

The Lu-Chipman decomposition describes the Mueller matrix as a product of three 4 × 4 real matrices separating the main polarimetric information encoded in **M**,

3 $$ {\varvec{M}} = {\varvec{M}}_{\Delta } {\varvec{M}}_{R} {\varvec{M}}_{D} , $$

where ***M***_*Δ*_ represents a depolarizer, ***M***_*R*_, a generalized retarder, and ***M***_*D*_ is a generalized diattenuator. These matrices can be used to obtain the values of the linear and circular retardance and the linear and circular dichroism. Moreover, the orientation of the axis defining linear retardance and dichroism can also be obtained from matrices ***M***_*R*_ and ***M***_*D,*_ respectively. The details about the implementation of the Lu-Chipman decomposition have been largely discussed in the literature and are included in the extended information section for reader’s convenience.

To characterize the depolarizing content of the botanical samples, in this study it is discussed the use of a full depolarization space instead of a single observable. A depolarization space is an abstract mathematical space made of three or more depolarization-related metrics which are not fully independent but related to each other. A depolarization space gives information not just on how much light is depolarized but also on how it is depolarized by the sample. The definition of a depolarization space is not unique^52,53^ and a choice must be done based on multiple criteria such as discrimination power between depolarization metrics, computation time, adequacy to the physical problem treated among others^54^. The depolarization space used in this work is composed by the IPPs^55^, which can be directly deduced from the measured Mueller matrix of the sample. The set of IPPs is composed of three real magnitudes labelled as *P*_*1*_, *P*_*2*_, and *P*_*3*_ (with values from 0 to 1 each) defined as respective combinations of the four eigenvalues (taken in decreasing order *λ*_*0*_ ≥ *λ*_*1*_ ≥ *λ*_*2*_ ≥ *λ*_*3*_) of the covariance matrix **H** which is associated with the Mueller matrix, **M**^55^.

4 $$ P_{1} \equiv \frac{{\lambda_{0} - \lambda_{1} }}{{tr{\varvec{H}}}},P_{2} \equiv \frac{{\lambda_{0} + \lambda_{1} - 2\lambda_{2} }}{{tr{\varvec{H}}}},P_{3} \equiv \frac{{\lambda_{0} + \lambda_{1} + \lambda_{2} - 3\lambda_{3} }}{{tr{\varvec{H}}}}. $$

IPP parameters are restricted by the following inequalities^55^,

5 $$ 0 \le P_{1} \le P_{2} \le P_{3} . $$

The idea behind IPPs is that the response of any depolarizer can be synthesized as the incoherent sum of four components with different weights, which are regulated by the IPPs^50,56^. Accordingly, *P*_1_ is associated with the relative portion of a non-depolarizing component, P_3_ with the portion that is not fully depolarized, and *P*_2_*–P*_1_ measures the relative portion of a parallel component composed of an equiprobable mixture of two non-depolarizing elements^50^. In this context, IPPs allows for the differentiation between different types of depolarizers^56,57^, or, in other words, between different types of depolarization mechanisms, which may unveil differences among the structures and organs in the sample tissue. In contrast to the IPP, which provide complete quantitative information of depolarization, the depolarization index *P*_*∆*_^58^, commonly used in the polarimetric community, only provides an overall measure of the depolarizing power of the sample. Note that *P*_*∆*_ can eventually be calculated from the IPPs as^55^,

6 $$ P_{\Delta } = \frac{1}{\sqrt 3 }\sqrt {2P_{1}^{2} + \frac{2}{3}P_{2}^{2} + \frac{1}{3}P_{3}^{2} } . $$

### Optical microscope

The optical microscope is the same used in the polarimetric microscope (described below), but without the corresponding PSG and PSA.

### Polarimetric microscope

Polarimetric images (Figs. 2, 3 and 4) were obtained with a multimodal microscope polarimeter. The multimodal microscope is an innovative polarimetric imaging system that can be operated in two imaging modes, the real plane, and the Fourier plane (also called conjugate space plane). In real plane imaging mode, the microscope produces images of the studied sample, while in Fourier imaging mode the images correspond to the angular distribution of light transmitted or scattered by the sample. The instrument is coupled to a white light LED as a source, followed by a narrow-band spectral filter centred at a wavelength of 533 nm with a spectral width of 15 nm. The microscope is mounted in transmission configuration; the sample is located between two identical microscope objectives (one for imaging and another for illumination). The microscope objectives can be selected among different magnifications; 50×, 20×, or 5 × depending on the needs of a specific resolution and a numerical aperture of a desired image.

Thanks to the use of a series of relay lenses, it is possible to create conjugates of the back-focal planes (BFP) of the illumination and imaging objectives the illumination and imaging arms, respectively. The eventual insertion of pinholes in the conjugates of the BFP of the two objectives allows for the control of the direction and the aperture of the illuminating and the imaging beam, respectively. A pinhole (500 µm diameter) is used to illuminate the samples with a beam in normal incidence with an aperture of 5°. No pinhole is inserted in the imaging arm to maximize the collection of scattered light intensity and thus to optimize depolarization sensitivity of the measurements. The relay lens system provides a conjugate of the sample plane in both; the illumination and the imaging arms, therefore, the use of pinholes in those planes, helps to define the shape and size of the illuminated and imaged field of view (FOV) respectively. The insertion of a Bertrand lens in the optical path of the microscope allows for an easy switch between the real and the Fourier imaging modes^59^. In the present work only images in the real plane were used. The same instrument has been applied in the past to characterize the dependence of polarization and depolarization properties as function of sample thickness^60^ and digital histology of human samples to study the influence of sample thickness of polarimetric observables^61^.

### Fluorescence microscope

Fluorescence images are obtained by a commercial *Olympus Fluoview 1000* microscope. The system can illuminate the sample with six different wavelengths and a multiple combination of them. Among all the available wavelengths, three are generated with diode lasers (405, 559 and 635 nm) and the other three are generated with a multiline argon laser (458, 488, and 515 nm). The fluorescence images presented in this work were acquired using three filters (Olympus U-MWU2, U-MNB2, U-MWG2) which are centred at 330 nm, 470 nm, and 510 nm, respectively. The equipment allows for the acquisition of images in four channels simultaneously with a resolution of 200 nm, using a spectral detection system. The signal can be captured by three photomultipliers (PMT) for fluorescence and/or reflection, plus an external detector for transmitted light. Captions can be made in 5 dimensions, three for space, one for time, and one for wavelength (xyztλ). The control of CO_2_ delivery and temperature allows the system to carry out in-vivo experiments over time.

### Phase contrast microscope

The phase contrast microscope is the same instrument used to measure the fluorescence response (the Olympus Fluoview 1000 microscope) but equipped with two phase contrast elements: a phase contrast condenser annulus located under the microscope stage, and a phase ring placed above the objective.

### Electron microscope

The observations were made with a scanning electron microscope (*Jeol J7100F*) of the Centros Científicos y Tecnológicos de la Universidad de Barcelona (CCiT). The preparation of the leaves before observation was performed as follows. They were dehydrated with alcohols of increasing graduation until the absolute alcohol. Afterwards, they were brought to the critical point and were coated with a special carbon thin film deposited using thermal evaporation. The resolution of the electron microscope is of 100–500 nm depending on the selected magnification.

## Supplementary Information

Supplementary Information.
