# Supplementary material for: Polarimetric imaging microscopy for advanced inspection of vegetal tissues
Source: Sci Rep. 2021 Feb 16;11:3913. doi: 10.1038/s41598-021-83421-8 (PMC7887219; doi:10.1038/s41598-021-83421-8)
Supplement: Supplementary file 1 — Supplementary Information. [file 41598_2021_83421_MOESM1_ESM.pdf]

Polarimetric imaging microscopy for advanced inspection of vegetal tissues

Albert Van Eeckhout,[1,*] Enrique Garcia-Caurel,[2] Teresa Garnatje,[3] Juan Carlos Escalera,[1] Mercè Durfort,[4] Josep Vidal,[1] José J. Gil,[5] Juan Campos,[1] Angel Lizana[1]

[1] Grup d'Òptica, Physics Department, Universitat Autònoma de Barcelona, 08193, Bellaterra, Spain

[2] LPICM, CNRS, Ecole polytechnique, Institut Polytechnique de Paris, 91120 Palaiseau, France

[3] Botanical Institute of Barcelona (IBB, CSIC-ICUB), 08038, Barcelona, Spain

[4] Departament de Biologia Cel·lular, Fisiologia & Immunologia. Facultat de Biologia, Universitat de Barcelona, 08028, Barcelona, Spain

[5] Department of applied physics, University of Zaragoza, Pedro Cerbuna 12, 50009, Zaragoza, Spain

*albert.vaneeckhout@uab.cat

Supplementary Information

The Lu-Chipman product decomposition

The Lu-Chipman decomposition is a product decomposition which describes the Mueller matrix, $\boldsymbol{M}$, as a concatenation of three matrices separating the main polarimetric information encoded in $\boldsymbol{M}$.

$$\boldsymbol{M} = \boldsymbol{M}_{\Delta}\boldsymbol{M}_{R}\boldsymbol{M}_{D}, \tag{a1}$$

where $\boldsymbol{M}_{\Delta}$ is the Mueller matrix associated to a depolarizer (with nonzero polarizance in general), $\boldsymbol{M}_{R}$ is the Mueller matrix of a pure retarder and $\boldsymbol{M}_{D}$ is the Mueller matrix of a pure diattenuator.

The matrix of a diattenuator can in general be written as:

$$\boldsymbol{M}_{D} = \begin{pmatrix} 1 & \boldsymbol{D}^{T} \\ \boldsymbol{D} & \boldsymbol{m}_{D} \end{pmatrix}. \tag{a2}$$

The diattenuation vector, $\boldsymbol{D}$, is defined from the first row of the original matrix $\boldsymbol{M}$ as: $\boldsymbol{D} = \frac{1}{M_{\{1,1\}}}(M_{\{1,2\}} \quad M_{\{1,3\}} \quad M_{\{1,4\}})$. Values between {} indicate the corresponding matrix element index. The 3x3 submatrix $\boldsymbol{m}_{\boldsymbol{D}}$ is determined by the diattenuation vector as follows:

$$\boldsymbol{m}_{D} = a\boldsymbol{I}_{\boldsymbol{3}} + b\boldsymbol{D}\boldsymbol{D}^{T} \quad \text{with} \quad a = \sqrt{1 - D^{2}} \quad \text{and} \quad b = {}^{1-a}/_{D^{2}}, \tag{a3}$$

where $\boldsymbol{I}_{3}$ is the 3x3 identity matrix, and D is the absolute value of the diattenuation vector $\boldsymbol{D}$.

The multiplication of the original Mueller matrix by the inverse of $\boldsymbol{M}_{\boldsymbol{D}}$, is needed to obtain a new matrix $\boldsymbol{M'}$ without the effect of the diattenuator. The expression of the inverse of $\boldsymbol{M}_{\boldsymbol{D}}$ can be expressed in a close form as follows:

$$\boldsymbol{M}_{\boldsymbol{D}}^{-\boldsymbol{1}} = \frac{1}{a^{2}}\begin{pmatrix} 1 & -\boldsymbol{D}^{T} \\ -\boldsymbol{D} & \boldsymbol{I}_{\boldsymbol{3}} \end{pmatrix} + \frac{1}{a^{2}(a+1)}\begin{pmatrix} 1 & \boldsymbol{0}^{T} \\ \boldsymbol{0} & \boldsymbol{D}\boldsymbol{D}^{T} \end{pmatrix}. \tag{a4}$$

When multiplied by the original Mueller matrix gives:

$$\boldsymbol{M}' = \boldsymbol{M}\boldsymbol{M}_{\boldsymbol{D}}^{-\boldsymbol{1}} = \begin{pmatrix} 1 & \boldsymbol{D}^{T} \\ \boldsymbol{P} & \boldsymbol{m} \end{pmatrix}\boldsymbol{M}_{\boldsymbol{D}}^{-\boldsymbol{1}} = \begin{pmatrix} 1 & \boldsymbol{0}^{T} \\ \frac{\boldsymbol{P}-\boldsymbol{m}\boldsymbol{D}}{a^{2}} & \boldsymbol{m}' \end{pmatrix}. \tag{a5}$$

In analogy with the diattenuation vector, the polarizance vector, $\boldsymbol{P}$, can also be defined from the first column of the original Mueller matrix as: $\boldsymbol{P} = \frac{1}{M_{\{1,1\}}}(M_{\{2,1\}} \quad M_{\{3,1\}} \quad M_{\{4,1\}})$. The matrix, $\boldsymbol{m}$, corresponds to the 3x3 bottom-right submatrix in the original Mueller matrix.

In turn, the general expressions for the matrices $\boldsymbol{M}_{\Delta}$, $\boldsymbol{M}_{R}$ are the following:

$$\boldsymbol{M}_{\boldsymbol{\Delta}} = \begin{pmatrix} 1 & \boldsymbol{0}^{T} \\ \boldsymbol{P}_{\boldsymbol{\Delta}} & \boldsymbol{m}_{\boldsymbol{\Delta}} \end{pmatrix} \quad \text{and} \quad \boldsymbol{M}_{\boldsymbol{R}} = \begin{pmatrix} 1 & \boldsymbol{0}^{T} \\ \boldsymbol{0} & \boldsymbol{m}_{\boldsymbol{R}} \end{pmatrix} \tag{a6}$$

Therefore, the auxiliary matrix $\boldsymbol{M'}$ can be written as:

$$\boldsymbol{M}' = \begin{pmatrix} 1 & \boldsymbol{0}^T \\ \boldsymbol{P_\Delta} & \boldsymbol{m_\Delta} \end{pmatrix} \begin{pmatrix} 1 & \boldsymbol{0}^T \\ \boldsymbol{0} & \boldsymbol{m_R} \end{pmatrix} = \begin{pmatrix} 1 & \boldsymbol{0}^T \\ \boldsymbol{P_\Delta} & \boldsymbol{m_\Delta m_R} \end{pmatrix}. \quad \text{(a7)}$$

And the following definitions can be stablished: $\boldsymbol{P_\Delta} = \frac{\boldsymbol{P - mD}}{1-D^2}$, and $\boldsymbol{m}' = \boldsymbol{m_\Delta m_R}$

The submatrix $\boldsymbol{m_\Delta}$ can be obtained from the product $\boldsymbol{m}'(\boldsymbol{m}')^{\boldsymbol{T}}$ and the corresponding eigenvalues, λ_1, λ_2, and λ_3, of the latter matrix product as follows:

$$\boldsymbol{m_\Delta} = \pm(\boldsymbol{m}'(\boldsymbol{m}')^{\boldsymbol{T}} + k_2\boldsymbol{I_3})^{-1}(k_1\boldsymbol{m}'(\boldsymbol{m}')^{\boldsymbol{T}} + k_3\boldsymbol{I_3}), \quad \text{(a8)}$$

with: $k_1 = \sqrt{\lambda_1} + \sqrt{\lambda_2} + \sqrt{\lambda_3}$, $k_2 = \sqrt{\lambda_1\lambda_2} + \sqrt{\lambda_2\lambda_3} + \sqrt{\lambda_1\lambda_3}$, and $k_3 = \sqrt{\lambda_1\lambda_2\lambda_3}$.

The sign + or - in Eq. (a8) is the same as that of the determinant of the matrix ***m'***. With that, it is possible to determine the submatrix $\boldsymbol{m_R}$ as follows:

$$\boldsymbol{m_R} = \boldsymbol{m_\Delta^{-1}}(\boldsymbol{m}')^{\boldsymbol{T}} = \pm(k_1\boldsymbol{m}'(\boldsymbol{m}')^{\boldsymbol{T}} + k_3\boldsymbol{I_3})^{-1}(\boldsymbol{m}'(\boldsymbol{m}')^{\boldsymbol{T}}\boldsymbol{m}' + k_2\boldsymbol{m}'). \quad \text{(a9)}$$

The sign here also depends on the sign of the determinant of the matrix ***m'***.

The total retardance R (combination of linear and circular retardance) can be calculated by using the following expression.

$$R = cos^{-1}\left[\frac{tr(\boldsymbol{M_R})}{2} - 1\right] \quad \text{(a10)}$$

Further analysis of $\boldsymbol{M_R}$ can be accomplished by decomposing it as the product of a linear retarder and circular retarder, i.e., $\boldsymbol{M_R} = \boldsymbol{M_{LR}} \cdot \boldsymbol{M_{CR}}$ as follows:

$$\boldsymbol{M}_R = \begin{pmatrix} 1 & 0 & 0 & 0 \\ 0 & cos^2 2\theta + sin^2 2\theta\, cos\delta & sin2\theta\, cos2\theta(1-cos\delta) & -sin2\theta sin\delta \\ 0 & sin2\theta cos2\theta(1-cos\delta) & sin^2 2\theta + cos^2 2\theta\, cos\delta & cos2\theta sin\delta \\ 0 & sin2\theta\, sin\delta & cos2\theta sin\delta & cos\delta \end{pmatrix} \cdot \begin{pmatrix} 1 & 0 & 0 & 0 \\ 0 & cos2\psi & sin2\psi & 0 \\ 0 & -sin2\psi & cos2\psi & 0 \\ 0 & 0 & 0 & 1 \end{pmatrix} \quad \text{(a11)}$$

The first matrix corresponds to a linear retarder with linear retardance, δ, which optic axis is rotated by and angle, θ, respect to a given reference. The second matrix represents a circular retarder with circular retardance, 2ψ, which induces a rotation of an angle, ψ, when it is illuminated with linearly polarized light. The optical rotation, ψ and the linear retardance, δ, can be determined as follows:

$$\psi = \tan^{-1}([\boldsymbol{M_{R\{2,1\}}} - \boldsymbol{M_{R\{1,2\}}}]/[\boldsymbol{M_{R\{1,1\}}} - \boldsymbol{M_{R\{2,2\}}}]) \quad \text{(a12)}$$

$$\delta = \cos^{-1}\left(\sqrt{[\boldsymbol{M_{R\{1,1\}}} + \boldsymbol{M_{R\{2,2\}}}]^2 + [\boldsymbol{M_{R\{2,1\}}} - \boldsymbol{M_{R\{1,2\}}}]^2} - 1\right) \quad \text{(a13)}$$

Note that the Eqs. (a12) and (a13) are independent of the product order between the linear retarder and the circular retarder in Eq. (a11).

The optical rotation angle, ψ, the Mueller matrix of the linear retarder, $\boldsymbol{M_{RL}}$, can be obtained as follows:

$$M_{LR} = M_R \cdot M_{CR}^{-1}(\psi) \quad \text{(a14a)} \qquad \text{or} \qquad M_{LR} = M_{CR}^{-1}(\psi) \cdot M_R \quad \text{(a14b)}$$

In general, the value of $\boldsymbol{M}_{RL}$ strongly depends on the choice between Eq. (a14a) and Eq. (a14b), therefore, care must be taken when an automatic implementation of the above equations is used. In the present work the measured optical rotation is negligible, thus giving no relevance to the combination order.

The angle θ of the linear retarder can be obtained from the $\boldsymbol{M}_{RL}$ matrix, as follows:

$$\theta = 0.5\, tan^{-1}(r_2/r_1), \tag{a15}$$

where,

$$r_i = (1/2\, sin\delta) \sum_{j,k=1}^{3} \varepsilon_{jk} \boldsymbol{m}_{LR_{jk}} \tag{a16}$$

and ε_{jk} is the Levi-Cita permutation symbol and $\boldsymbol{m}_{LR}$ is the 3x3 bottom-right submatrix in the $\boldsymbol{M}_{LR}$ matrix.

Summary table of polarimetric metrics

To summarize the different parameters used in the manuscript to obtain the distinct polarimetric images, we have grouped them into the following table. The table contains the parameter name in the left column and the corresponding description in the adjacent columns to the right. The colour code groups polarimetric metrics by physical typology.

**Mueller matrix**	$\boldsymbol{M} = \begin{pmatrix} m_{00} & m_{01} & m_{02} & m_{03} \\ m_{10} & m_{11} & m_{12} & m_{13} \\ m_{20} & m_{21} & m_{22} & m_{23} \\ m_{30} & m_{31} & m_{32} & m_{33} \end{pmatrix} = m_{00} \begin{pmatrix} 1 & \boldsymbol{D}^T \\ \boldsymbol{P} & \boldsymbol{m} \end{pmatrix}$	Transfer function of a polarimetric system.
**Diattenuation**	$D = \|\boldsymbol{D}\| = \|m_{01} + m_{02} + m_{03}\|$	Measures the transmittance dependence with input polarization.
**Linear Retardance**	$\delta = cos^{-1}\left(\left\{\left[M_{R_{11}} + M_{R_{22}}\right]^2 + \left[M_{R_{21}} - M_{R_{12}}\right]^2\right\}^{1/2} - 1\right)$	Measures the phase difference introduced to the orthogonal components of an input beam.
**Fast axis angle**	$\theta = 0.5\, tan^{-1}(r_2/r_1), r_i = (1/2\, sin\delta) \sum_{j,k=1}^{3} \varepsilon_{jk} \boldsymbol{m}_{LR_{jk}}$	Measures the optical axis orientation.
**IPP parameters (Depolarization)**	$P_1 \equiv \frac{\lambda_0 - \lambda_1}{tr\boldsymbol{H}}$ $\quad P_2 - P_1 \equiv \frac{\lambda_1 - \lambda_2}{tr\boldsymbol{H}}$	Measures the sample randomness.

Visibility contrast of raphides and stomata

In order to quantify the contrast of raphides and stomata we propose the evaluation of the visibility parameter:

$$V = \frac{|\bar{I}_{str} - \bar{I}_{back}|}{\bar{I}_{str} + \bar{I}_{back}} \tag{a17}$$

with $\bar{I}_{str}$ and $\bar{I}_{back}$ being the average signal intensities of the analyzed structure and the background cells, respectively. Visibility values are between 0 (null contrast) and 1 (maximum contrast) and all the calculated visibilities are presented in Table S1. In the case of Figs. 2(a)-(d), 4(a)-(b) and 5(b) we study the visibility of raphides with respect to the surrounding background. To calculate the average intensity of the raphide we selected regions of 21x21 pixels in size highlighted with a red square in Figs. 2(c), 4(b) and 5(b). Analogously, we used background cell regions of size 21x21 pixels, which are represented with a green square in Figs. 2(c), 4(b) and 5(b), to calculate the average intensity of the background. The average intensity values of the raphide and background regions are summarized in Table S1.

In addition to the visibility related to the presence of raphides for different observables, the main manuscript also provides the visibility value of a stoma that is imaged with a phase contrast microscope (Fig. 5(a)). The regions used to calculate the visibility of the stoma are 21x11 pixels in size, and they are delimited with a purple rectangle and a yellow rectangle, respectively. The purple rectangle is associated with the stomata intensity and the yellow one with the background cells. These average intensity values are also provided in Table S1.

The visibility parameter gives a quantification of the image contrast. However, this parameter does not consider the evaluation of the eventual noise present in the measurements. Accordingly, we propose the use of the standard deviation within each selected region, which is compared with the $\bar{I}_{str} - \bar{I}_{back}$ difference. This new parameter is calculated using the following equation:

$$s = \frac{\sigma}{|\bar{I}_{str} - \bar{I}_{back}|} = \sqrt{\frac{\sum_i^N (I_i - \bar{I})^2}{(N-1)|\bar{I}_{str} - \bar{I}_{back}|^2}} \tag{a18}$$

where N is the number of pixels of each region of interest. The interpretation of the values of s is as follows: if s is higher than 1, fluctuations within the area of interest are higher than $\bar{I}_{str} - \bar{I}_{back}$ difference and therefore the studied structure is difficult to distinguish from the background in terms of contrast. On the contrary, values of s lower than one mean that the studied structure appears to be distinct than the surrounding fluctuations. Therefore, the lower the value of s is, the sharper the contrast between the studied object and the background.

The analysis of different s (Table S1) shows that all the visibilities are comparable as the corresponding s values are similar (from 0.5 to 0.25) except in the case of the regular intensity measurement. In this case s values are higher than 1 which means that the studied structures are difficult to distinguish. Therefore, the latter result is in line with the visibility result, which was close to 0, also indicating that the structure is low contrasted.

Table S1. Intensity average, visibility and standard deviation compared with the $\bar{I}_{str} - \bar{I}_{back}$ gap of the studied structures that are imaged in Figs. 2(a)-(d), 4(a)-(b) and 5(a)-(b).

	Focused raphide				Defocused raphide		Phase contrast	
	Fig.2(a)	Fig.2(b)	Fig.2(c)	Fig.2(d)	Fig.4(a)	Fig.4(b)	Fig.5(a)	Fig.5(b)
	Intensity	P_Δ	P_1	P_2-P_1	Intensity	P_2-P_1	stoma	raphide
$\bar{I}_{str}$	1.47	0.19	0.12	0.14	0.92	0.11	55.3	56.4
$\bar{I}_{back}$	1.39	0.31	0.29	0.02	0.93	0.02	124	74.8
V	0.03	0.24	0.41	0.72	0.00	0.67	0.38	0.14
s_{str}	0.38	0.07	0.04	0.08	2.24	0.10	0.08	0.17
s_{back}	1.45	0.24	0.18	0.09	4.07	0.08	0.07	0.22

Hedera helix and *Vitis vinifera* polarimetric images

The manuscript entitled "Polarimetric imaging microscopy for advanced inspection of vegetal tissues" highlights the interest of polarimetric microscopy to study an *Epipremnum aureum* leaf, but the suitability of polarimetric methods there discussed is also observed in other plant specimens as *Hedera helix*, and *Vitis vinifera*. In the following, we provide microscopic images of these two specimens to illustrate the suitability of such polarimetric method to study other specimens. First, we show the image results concerning the measurements of a small section of a *Vitis vinifera* leaf (Fig. S1). The region imaged in Fig. S1 corresponds to the obverse face of the leaf pointing to the imaging microscope objective. The region comprises two veins that are clearly observed in the regular intensity channel (Fig. S1(a)) and in the P_1 channel (Fig. S1(b)). Likewise, the cell walls that are present in the leaf are also clearly seen in both images. Conversely, some elliptically shaped structures, represented in black in Fig. S1(b), are undistinguishable in the regular intensity image (Fig. S1(a)). These structures are invisible in intensity images as their response in that channel is very similar to the one produced by their neighbour structures. Alternatively, they can be detected by using the P_1 observable as they depolarize light in a different way concerning the surroundings. Note that thanks to the anisotropic depolarizing behaviour of such structures, they are highlighted in the P_2-P_1 channel (Fig. S1(c)). This polarimetric response is like the one

obtained with the raphides contained in the *Epipremnum aureum* leaf analysed in the main manuscript. Although we do not have a solid evidence at the moment of writing the present manuscript, we hypothesize that the structures may correspond to either solid crystals (like raphides) within the leaf, or they may be microscopic parasites or infection present in the leaf. The proper identification of these structures will be performed in a future study.

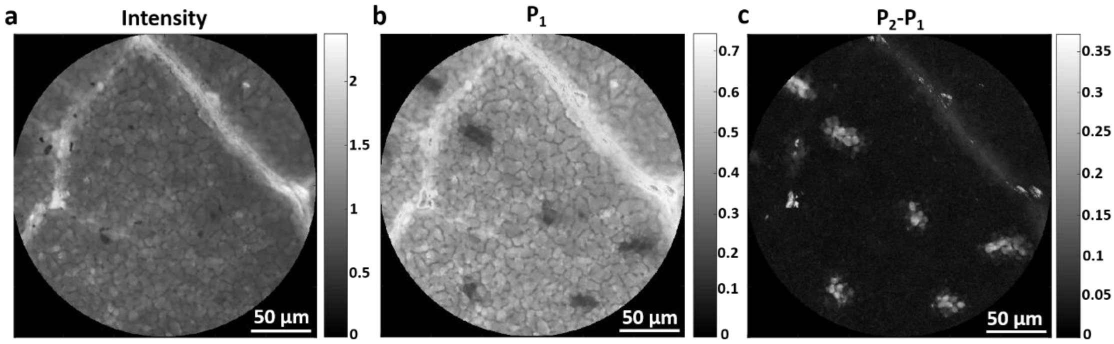

Figure S1. Polarimetric image of a small section of an *Vitis vinifera* leaf comprising two veins. The region is imaged by using the (a) Intensity, (b) P_1, and (c) P_2-P_1 channels.

As a second example, we show the images resulting from measuring a *Hedera helix* leaf (Fig. S2) with the same Mueller matrix microscope. The leaf was measured with the lower face of the leaf pointing to the imaging microscope objective. In the intensity image of the *Hedera helix* leaf (Fig. S2 (a)) we can observe a vein crossing the image from the bottom to the left, and a group of star-like structures with 4 to 6 arms. The star-like structures are called trichomes and their key role is attaching the leaves to different solid substrates. The analysis of Fig. S2 (a) shows that some of the trichomes, e.g., the seen in the top of the image, are difficult to distinguish because they are out of focus. However, the same trichomes can be clearly distinguished in Fig. S2 (b) thanks to its birefringent properties. Fig. S2 (b) is a pseudocoloured image that includes the linear retardance information of the sample (encoded in the brightness of the image colours), and the fast axis orientation (represented with assorted colours). It can be observed that for each arm of the imaged trichomes, the orientation of the retardance is parallel to the long axis of the corresponding arm. Further studies will analyse the origin of that birefringent behaviour and possible relations between the measured birefringence and the presence of either mechanical stress or oriented polymers in the arm tensions.

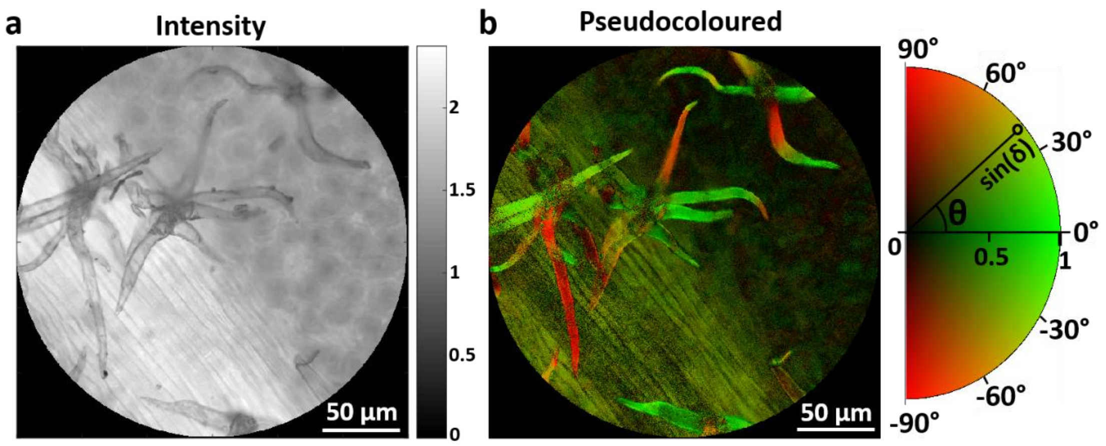

Figure S2. (a) Intensity image and (b) pseudocoloured image of a collection of trichomes of a *Hedera helix* leaf. The pseudocoloured image comprises the linear retardance information of the sample (shown into a white-black scale; radius of the semi-circular colour scale), and the fast axis orientation (represented with different colours; perimeter of the semi-circular colour scale).
